# Cerebral blood flow velocity progressively decreases with increasing levels of verticalization in healthy adults. A cross-sectional study with an observational design

**DOI:** 10.3389/fneur.2023.1149673

**Published:** 2023-04-17

**Authors:** Julian Deseoe, Anne Schwarz, Theodor Pipping, Aurelia Lehmann, Janne M. Veerbeek, Andreas R. Luft, Susanne Wegener, Christoph Globas, Jeremia P. O. Held

**Affiliations:** ^1^Department of Neurology, University Hospital Zurich and University of Zurich, Zurich, Switzerland; ^2^Neurocenter, Luzerner Kantonsspital, Lucerne, Switzerland; ^3^Rehabilitation Center Triemli Zurich, Valens Clinics, Zurich, Switzerland

**Keywords:** blood pressure, cerebral blood flow velocity, healthy, ultrasound, verticalization

## Abstract

**Background:**

Autoregulation of the cerebral vasculature keeps brain perfusion stable over a range of systemic mean arterial pressures to ensure brain functioning, e.g., in different body positions. Verticalization, i.e., transfer from lying (0°) to upright (70°), which causes systemic blood pressure drop, would otherwise dramatically lower cerebral perfusion pressure inducing fainting. Understanding cerebral autoregulation is therefore a prerequisite to safe mobilization of patients in therapy.

**Aim:**

We measured the impact of verticalization on cerebral blood flow velocity (CBFV) and systemic blood pressure (BP), heart rate (HR) and oxygen saturation in healthy individuals.

**Methods:**

We measured CBFV in the middle cerebral artery (MCA) of the dominant hemisphere in 20 subjects using continuous transcranial doppler ultrasound (TCD). Subjects were verticalized at 0°, −5°, 15°, 30°, 45° and 70° for 3–5 min each, using a standardized Sara Combilizer chair. In addition, blood pressure, heart rate and oxygen saturation were continuously monitored.

**Results:**

We show that CBFV progressively decreases in the MCA with increasing degrees of verticalization. Systolic and diastolic BP, as well as HR, show a compensatory increase during verticalization.

**Conclusion:**

In healthy adults CBFV changes rapidly with changing levels of verticalization. The changes in the circulatory parameters are similar to results regarding classic orthostasis.

**Registration:**

ClinicalTrials.gov, identifier: NCT04573114.

## 1. Introduction

Cerebral blood flow (CBF) autoregulation is essential to the brain's energy supply and is dysfunctional in many diseases, including stroke or neurodegenerative disorders (1). In stroke, failure of CBF regulation is associated with worse functional outcome (2).

To understand autoregulation in varying body positions of healthy individuals, studies have used cerebral blood flow velocity (CBFV) monitoring (3, 4). These investigations did not use dynamic protocols, i.e., changing position several times with <10 min maintaining one. Therefore, these are difficult to transfer to routine clinical situations in which patients need to be mobilized out of bed within a short time. It is unknown how dynamic verticalization within 0° and 70° of head-up tilt and head-down tilt might affect CBFV, blood pressure (BP), heart rate (HR) and oxygen saturation in healthy adults.

Here we report CBFV regulation during dynamic changes of body position in a healthy cohort.

## 2. Methods

### 2.1. Setting and participants

This study was a mono-center cross-sectional study with a prospective observational design. It was conducted in accordance with the protocol and approved by the cantonal ethics committee of Zurich (BASEC-Nr. 2020-01732). The study was prospectively registered at ClinicalTrials.gov (Identifier: NCT04573114). The study was conducted in accordance with the current Declaration of Helsinki. Twenty healthy subjects were recruited by flier and over personal contacts at the University Hospital Zurich. The participants had to be 18 years or older. Exclusion criteria were any preexisting conditions potentially affecting cerebral blood flow and/or temporal bone windows insufficient to enable ultrasound penetration.

We recorded age, sex, handedness, physical activities, height, weight, medications, and medical history of participants. All measurements were performed between 10/2020 and 10/2021 at the Department of Neurology, University Hospital Zurich, Switzerland.

### 2.2. Measurements

For the verticalization, a Sara Combilizer produced by Arjo was used (5). CBFV was measured with a Holter Transcranial Doppler (TCD) ultrasound over the Middle Cerebral Artery (MCA) of the dominant hemisphere. The method was adapted from Schaafsma et al. (6). A 1.5 MHz TCD probe (Transcranial Doppler Holter, Atys Medical, France) was used, and the MCA was insonated at a depth of 46–54 mm with a gate length of 8–11 mm. Systolic, diastolic, and average blood flow velocity were measured continuously. Average blood flow velocity per second was extracted for analysis. BP and HR were measured every minute with a wrist monitor (BC 58, Beurer, Germany) on the left arm. The left arm was kept at heart level during the protocol *via* an armrest. Oxygen saturation was continuously measured *via* a pulse oximetry clip (PO-100, Pulox, Germany) on the right index finger and recorded every minute.

#### 2.2.1. Verticalization protocol

**Figure 1:**
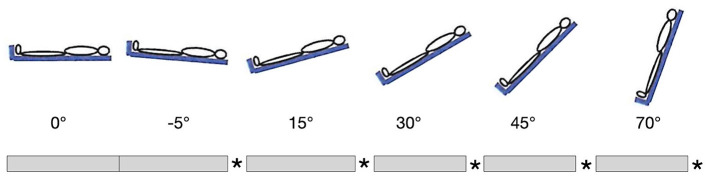
Verticalization protocol. Gray bars represent 3–5 min at indicated position, stars represent 3–5 min at 0°. The first 3–5 min at 0° were taken as baseline.

Healthy subjects were placed in supine position on the Sara Combilizer. BP and HR were measured at the wrist. The TCD was placed over the temporal bone of the dominant hemisphere by a skilled technician, and the measurement was started. Baseline CBFV was measured for 3–5 min at 0° tilt (horizontal position). Then subjects were positioned at −5° (head-down tilt), 15°, 30°, 45°, and 70° of tilt, in that order, with 3–5 min at every position. Between the positions, the subjects were always brought back to 0° for 3–5 min, which would allow us to detect any changes in baseline over time. Finally, after the 70° head-up tilt position, the subjects were brought to 0° for 3–5 min. Change of positions was done with a speed of ~2°/s. Participants were instructed to be as still as possible during measurements in order to limit artifacts. The verticalization protocol is shown in Figure 1.

Adverse events were recorded, and medical aid was available in the case of symptoms. Due to potential orthostatic reaction with the changes in position, expected possible adverse events were nausea, dizziness, headache and loss of consciousness.

The primary aim was to assess the association between progressive levels of verticalization and CBFV in the MCA. Secondary aims were to assess the association between progressive levels of verticalization and BP, as well as HR and oxygen saturation. In addition, we wanted to detect the impact of participant specific parameters on CBFV, which might have to be adjusted for when comparing this study population to others. These were age, body mass index (BMI) and sex.

### 2.3. Statistical analysis

A formal calculation of sample size was not necessary as this study is a pilot, observational study, where a sample size of 12 participants is recommended (7). We recruited 20 subjects to allow for an analysis including age, sex and BMI as covariates.

All statistical analysis was performed using RStudio software (version 1.2.5033) (8) with R version 3.6.3 (9). The mean CBFV, BP, HR and oxygen saturation was calculated for every position and participant, excluding the first minute in each position, to allow adjustment. Then, the mean and standard deviation of all participants for each position were calculated for CBFV, BP, HR and oxygen saturation. The first 3–5 min at 0° were taken as baseline. The differences between the phases at 0° were inspected for trends throughout the protocol to detect any potential measurement bias over time.

A linear mixed effects model was calculated for baseline and the progressive levels of verticalization, to assess the effect of age, sex, BMI and position on CBFV using the R lme4 package (10). Normality of the residuals was assessed using QQ-plots, and a Shapiro-Wilk test was performed using the R rstatix package (11).

In order to assess the distribution of the measurements of HR, BP and oxygen saturation, a Shapiro-Wilk test was performed for each parameter and each level of verticalization, and a QQ-plot was created. A repeated measures ANOVA with Greenhouse-Geisser correction for sphericity was performed for statistical testing. The effect of increasing levels of verticalization on BP, HR and oxygen saturation were assessed. Sphericity was tested using Mauchly's test for sphericity. Pairwise comparisons were done using paired *t*-tests with Bonferroni correction for multiple testing if the ANOVA returned a significant result (*p* < 0.05). The results of the *t*-tests for the difference between baseline and −5° are reported here. The statistical testing was done with the rstatix package (11). For graphical representation, the difference to baseline was calculated for each position and variable. Participants with missing data points were included in the analysis and the average for the position for said participant was calculated without the missing data points. There was a minimum of four measurements of systolic and diastolic BP, HR and oxygen saturation per position for each participant.

## 3. Results

Twenty healthy participants were screened and included in the study. All participants completed the protocol and were included in the analysis. In two participants, the TCD device lost proper positioning and recorded 0 cm/s of CBFV for 2–12 s. These datapoints were excluded from the analysis. For the seven participants who had been previously prescribed regular medication (four of which were under antihypertensive treatment), the medication was not paused prior to the measurements. None of the patients had physical disabilities. One participant had a pacemaker and was included in all analysis. Table 1 summarizes the characteristics of the participants.

**Table 1:** Characteristics of participants.

**All participants (*n*)**	**20**
Sex [female] (%)	9 (45)
Age [y] (min, max)	63.7 (45, 79)
Handedness [right] (%)	18 (90)
Median number of times participants are physically active per week (min, max)	2 (0.7)
Height [cm] (SD)	174 (8.1)
Weight [kg] (SD)	77 (11.5)
BMI [kg/m^2^] (SD)	25.6 (4.0)

BMI, Body mass index. For physical activity the number of times participants performed physical activity per week is given.

First, the intervals at 0° of inclination at the beginning of the protocol and between the positions with increasing angles of verticalization were inspected for CBFV, BP, HR and oxygen saturation. As can be seen in Table 2, all parameters remained similar at 0° inclination throughout the whole protocol.

**Table 2:** Mean of clinical parameters during verticalization (−5° to 70°) and the pauses (0°0.1 to 0°0.5).

	**Baseline**	**−5°**	**0°(1)**	**15°**	**0°(2)**	**30°**	**0°(3)**	**45°**	**0°(4)**	**70°**	**0°(5)**
CBFV [cm/s]	44.6 (11.2)	44.5 (11.2)	43.8 (11.4)	43.7 (11.1)	44.4 (10.9)	43.1 (10.1)	44.4 (11.1)	40.8 (10.1)	44.5 (11.0)	39.8 (9.6)	43.8 (10.9)
Systolic BP [mmHg]	135.2 (11.5)	128.6 (11.9)	130.0 (11.4)	133.2 (11.8)	130.3 (12.4)	135.2 (11.9)	131.1 (11.0)	137.7 (9.5)	131.0 (11.8)	136.6 (16.2)	134.6 (13.7)
Diastolic BP [mmHg]	77.8 (8.0)	74.1 (7.4)	75.8 (6.7)	80.1 (4.9)	76.0 (6.9)	84.4 (5.8)	74.9 (5.0)	85.8 (5.4)	75.8 (5.4)	84.6 (7.6)	76.8 (6.3)
HR [bpm]	68.1 (10.7)	66.5 (9.9)	65.6 (10.5)	65.9 (10.9)	64.9 (10.5)	67.9 (10.4)	63.4 (10.0)	71.6 (10.1)	63.2 (10.0)	76.6 (11.3)	64.0 (10.6)
Oxygen sat. [%]	96.1 (1.8)	95.8 (1.7)	95.6 (1.7)	95.7 (1.7)	95.4 (1.7)	95.6 (1.5)	95.4 (1.6)	95.7 (1.6)	95.3 (1.5)	95.5 (1.9)	95.8 (1.7)

Numbers in parentheses show standard deviation. Baseline is the first 3–5 min at 0°.

**Figure 2:**
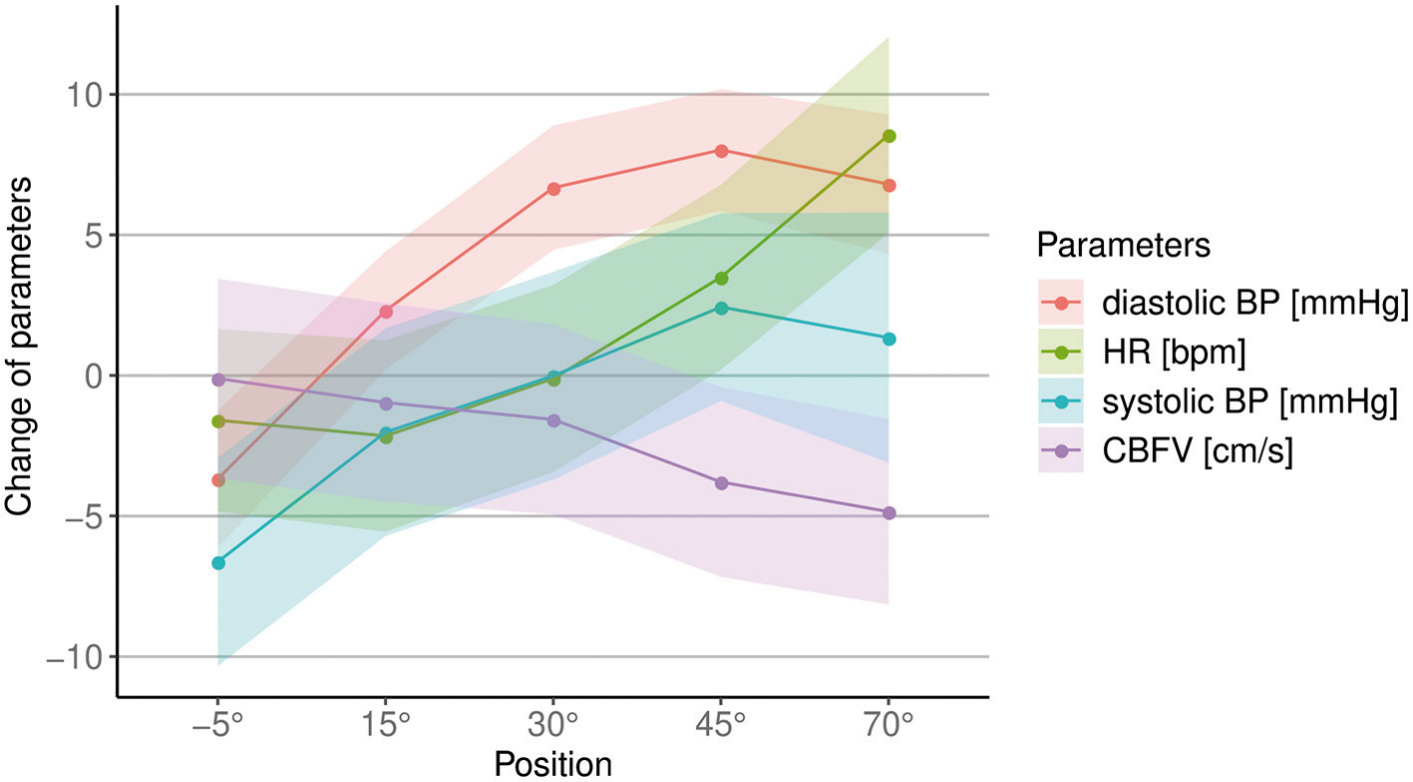
Overview of changes in cerebral blood flow velocity (CBFV), systolic and diastolic blood pressure (BP) and heart rate (HR) during different body positions. The unit for each parameter is shown in the legend. Points show mean difference to baseline, shaded areas show the standard error.

As there was no relevant change over time between measurements at 0° of inclination, only the difference to baseline at the increasing levels of verticalization are shown in all figures, and were considered in the analysis from this point forward, as we were primarily interested in the change of CBFV, BP, HR and oxygen saturation with increasing levels of verticalization. The first 3–5 min at 0° of inclination were always used as baseline. Figure 2 shows an overview over all circulatory parameters during verticalization.

**Figure 3:**
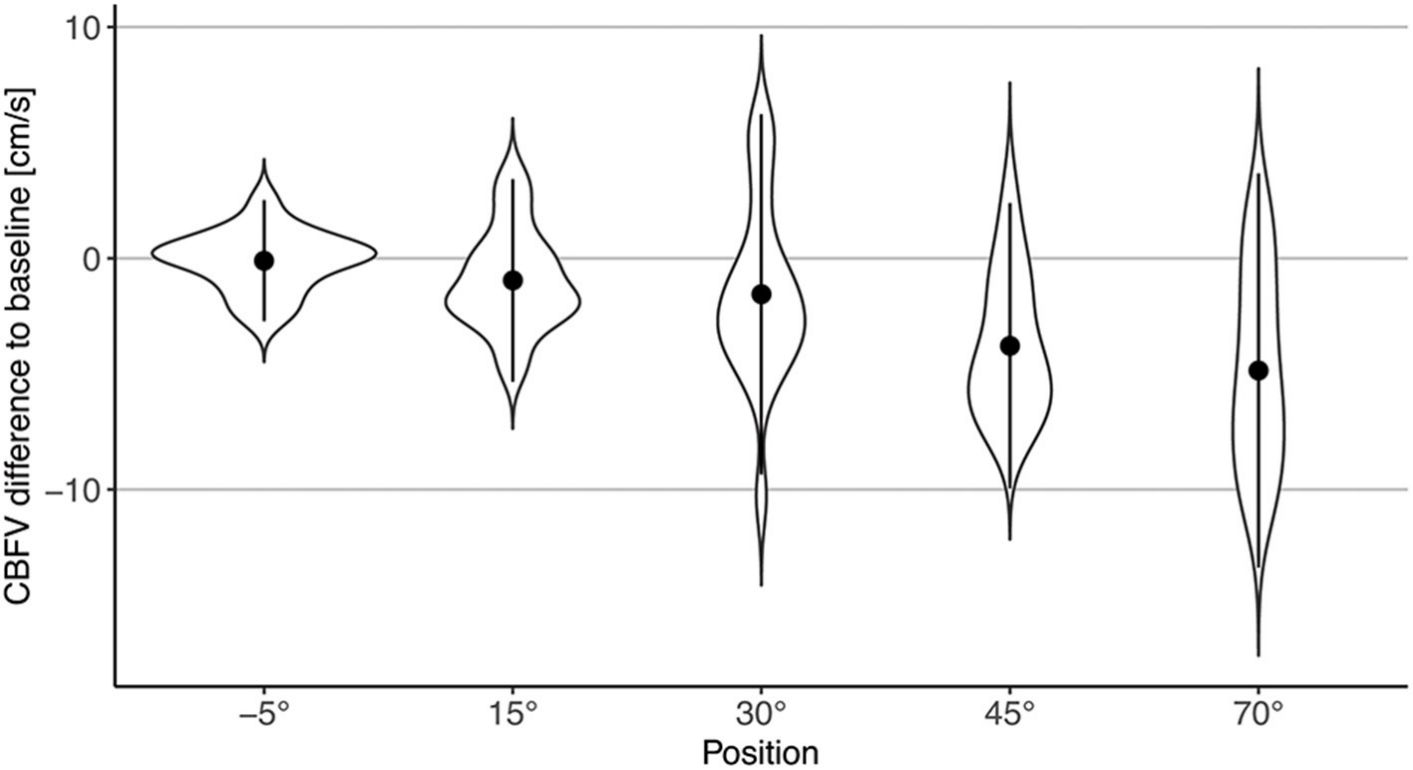
Violin plot of changes in CBFV during different body positions compared to baseline (0°). Points represent mean difference to baseline; error bars represent two standard deviations.

CBFV decreased progressively with increasing levels of head-up verticalization. The mean CBFV difference between consecutive head-up body positions ranged from −0.6 cm/s or −1.4% (difference between 15° and 30° of head-up tilt) to −2.2 cm/s or −5.2% (difference between 30° and 45° of head up tilt). CBFV did not change during head-down tilt. Change of CBFV is shown in Table 2 and Figures 2, 3. To quantify the impact of the positions as well as patient characteristics on CBFV, a linear mixed effects model was calculated. The linear mixed effects model is shown in Table 3.

**Table 3:** Linear mixed effects model for CBFV [cm/s].

	**Intercept**	**−5°**	**15°**	**30°**	**45°**	**70°**	**Sex (female)**	**Age [1/y]**	**BMI [m^2^/kg]**
Coefficient	46.68	−0.10	−0.96	−1.56	−3.78	−4.85	6.83	0.09	−0.42
95% CI [min/max]	−7.59/100.95	−1.43/1.23	−2.29/0.37	−2.89/−0.23	−5.11/−2.44	−6.18/−3.52	−3.34/17.00	−0.44/0.62	−1.69/0.85
*p*-value	0.11	0.88	0.16	0.02	<0.001	<0.001	0.21	0.74	0.52
Global *p*-value Positions	<0.001			

Position was encoded as 5 binary variables with baseline (0°) as reference category. Sex was also encoded as binary variable with male as reference category. Age and BMI were encoded as continuous variables. All these variables were included as fixed effects. No interactions were included in the model. The different participants were included as random effects. P-values were calculated using Satterwhaite's method. Residuals were assessed using a QQ-plot and found to be approximately normally distributed. The Shapiro-Wilk test provides no evidence that the residuals are not normally distributed (p = 0.23).

While changing positions clearly had an impact on CBFV, the data did not reveal any significant association of either sex, age, or BMI with CBFV.

**Figure 4:**
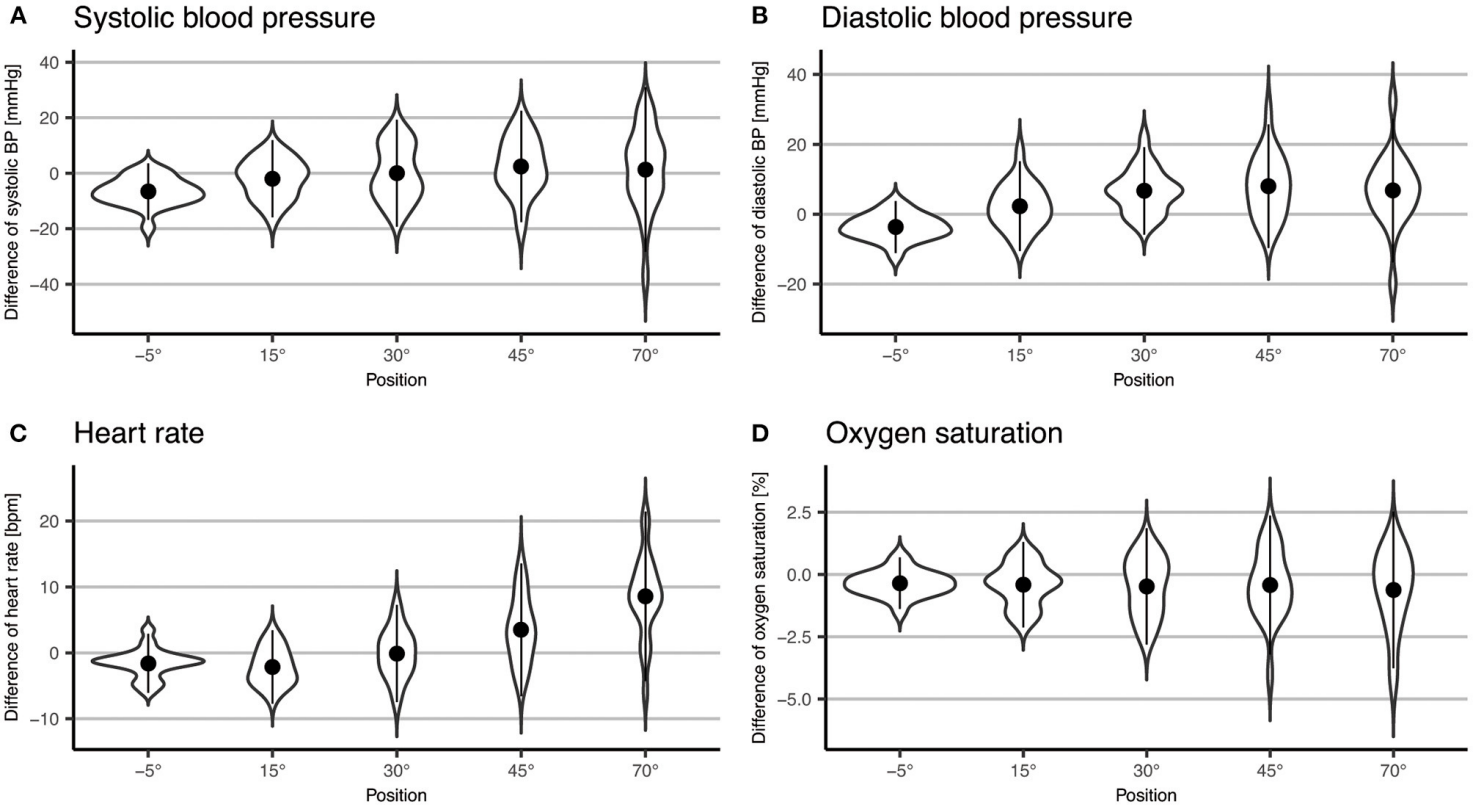
Violin plot of difference to baseline of systolic BP **(A)** and diastolic **(B)** blood pressure, heart rate **(C)** and oxygen saturation **(D)**. Points represent means; error bars represent two standard deviations.

We found that systolic BP changed significantly with increasing levels of verticalization (*p* = 0.03), with a mean increase 1.3 mmHg (±14.8 mmHg SD) from baseline to 70°. However, almost half of the participants showed a drop in systolic BP at 70° compared to baseline (Figure 4). Furthermore, diastolic BP changed significantly (*p* < 0.001). It increased by 6.8 mmHg (±10.3 mmHg SD) at 70° compared to baseline. HR also changed significantly (*p* < 0.001). It rose by 8.6 bpm (±6.4 bpm SD) from baseline to 70°. Oxygen saturation did not change significantly (*p* = 0.27). In addition, systolic BP (Δ = −6.6 mmHg ± 5.1 mmHg SD, *p* < 0.001), diastolic BP (Δ = −3.7 mmHg ± 3.7 mmHg SD, *p* = 0.004) and HR (Δ = −1.6 bpm ± 2.2 bpm, SD *p* = 0.08) all dropped at −5° compared to baseline. The results are shown in Table 4 and Figures 2, 4.

**Table 4:** Changes of systolic and diastolic blood pressure (BP), heart rate and oxygen saturation.

**Parameter**	**Change from baseline to 70°**	***p*-value mixed measures ANOVA**	**Greenhouse-Geisser correction**
Systolic BP (SD) [mmHg]	1.3 (14.8)	0.03	0.376
Diastolic BP (SD) [mmHg]	6.8 (10.3)	<0.001	0.403
Heart rate (SD) [bpm]	8.6 (6.4)	<0.001	0.378
Oxygen saturation (SD) [%]	−0.62 (1.6)	0.27	0.482

P-values are results of repeated measures Anova with Greenhouse-Geisser Correction for sphericity for overall change during increasing levels of verticalization. Sphericity was assessed using Mauchly's test for sphericity, which was significant (p < 0.001) for all parameters. The Shapiro-Wilk tests performed were mostly not significant at p = 0.05. The test was significant at −5° (p = 0.02) and 70° (p = 0.05) for systolic BP, significant at 45° (p = 0.03) and 70° (p = 0.04) for diastolic BP and significant at 70° (p = 0.002) for oxygen saturation. As most positions were non-significant and the deviations from the normal distribution are symmetric in the QQ-plots, there is little evidence that strong imprecision is introduced by assuming normality for statistical testing.

No serious adverse events occurred. One patient had symptoms of a brief orthostatic dysregulation at 70° tilt, with a drop in systolic BP below 90 mmHg for 3 min. This participant also reported headache and dizziness during −5° tilt, which completely recovered. Another participant reported mild headache and dizziness, without changes in BP and HR. The symptoms only lasted 1 min. One participant reported mild nausea in the first minute after being positioned to 45°.

## 4. Discussion

Our study shows that in healthy adults, there is a significant, progressive drop in CBFV with progressive levels of verticalization, using a dynamic protocol with 3–5 min maintaining single positions. Mean differences in CBFV between consecutive positions of head-up tilt ranged from −0.6 cm/s or −1.4% to −2.2 cm/s or −5.2%. At the same time, there was no relevant change in CBFV from baseline to −5°.

Systolic and diastolic BP both increased from baseline to 70°, with the change in diastolic BP being greater than the change in systolic BP. HR also increased from baseline to 70° position, while oxygen saturation remained stable during the progressive levels of verticalization. There was a drop in systolic and diastolic BP as well as HR at −5° of inclination compared to baseline. Some participants reported symptoms such as headache, dizziness and/or nausea. All these symptoms remitted after a maximum time of 3 min.

Overall, our results align well with existing literature. Selim et al. (4) found a decrease in CBFV in the MCA of healthy subjects from 0° compared to 70° of similar magnitude, although they measured CBFV for 10 min at each position. They did not measure CBFV at positions between 0° and 70°, which is a gap our study fills. Unlike in some other studies, we did not find an association of female sex with higher CBFV (3, 4, 12) or an association of higher BMI with lower CBFV (4, 12) in horizontal body position, possibly due to our smaller sample size. In several studies, higher age was significantly associated with lower CBFV (3, 4, 12), but we observed no such effect, most likely because the age range in our cohort was small (median = 65.5, IQR = 12.25, min = 45, max = 79).

There are several factors potentially contributing to the drop in CBFV with increasing levels of verticalization. One possible explanation could be that the diameter of the MCA increases, leading to a decreased CBFV while the CBF remains constant. It has however been shown that the diameter of the MCA does not change, when orthostasis is simulated by applying negative pressure to the lower extremities (13), making this explanation somewhat unlikely. Several studies have suggested a shift in cerebral blood flow autoregulation toward lower CBFV, following head-up tilt (14). One factor likely contributing to this, is small-vessel cerebral vasoconstriction during the orthostatic response (15). The mechanisms responsible for the drop in CBFV with increasing levels of verticalization, are however not completely understood (15).

Our results concerning BP and HR are close to what is expected for orthostatic reactions (16). The increase in both diastolic BP and HR is explained by a larger blood volume in the veins of lower extremities when standing up, compared to the supine position. This goes along with a sympathetic activation response, which leads to an increase in HR and vasoconstriction in the arterioles increasing diastolic BP. The expected increase with such orthostatic reaction is 10 mmHg for diastolic BP and 10–15 bpm for HR (16). The smaller increase in our study might be explained by the fact that verticalization was only performed up to 70° and not 90° and the fact that passive mobilization leads to less activation of the muscle compared to active mobilization. In theory, systolic BP remains constant during the orthostatic reaction. In our experiment, systolic BP changed significantly. However, systolic BP increased little compared to diastolic BP. Therefore, the changes in circulatory parameters during verticalization are like the theoretically expected changes during the normal orthostatic reaction.

The results of previous studies investigating the changes in HR and BP associated with changes in body positions were inconclusive. For systolic BP, results range from an increase in more upright body positions (17) to no significant change (18) to a significant decrease (19). Diastolic BP as well as HR did not change significantly in some studies (17, 19), while they increased in sitting position compared to supine in another (18). Hence, our findings concerning BP and HR during verticalization fall within the range of results of previous studies investigating BP and HR in different body positions (17–19). In summary, our study provides evidence that the circulatory changes during verticalization are comparable to the changes during the orthostatic reaction during conventional mobilization.

Using dynamic, passive verticalization to investigate CBF regulation has several advantages. Compared to conventional mobilization, the protocol can be standardized, e.g., without having to ask participants to stand up and without physical support by therapists. In addition, it allows CBFV measurements with continuous TCD, which would have been more prone to artifacts during conventional mobilization due to more bulk movement. Doppler ultrasound is widely used to measure CBFV (20). In addition, CBFV seems to be a good surrogate for CBF, at least during simulated orthostasis (13).

Our study has some limitations. For one, doppler ultrasound monitoring is prone to artifacts during verticalization. By averaging over multiple minutes per position and removing measurements during which the device recorded erroneous values for analysis, the effect of artifacts was minimized. Another limitation might be that end-tidal CO_2_ was not measured. Verticalization might induce hyperventilation in participants, leading to a cerebral vasoconstriction and thus lower CBFV (21). As end-tidal CO_2_ was not measured, we are not able to make a statement about the influence of this effect. However, according to our experience respiratory rate does not strongly increase during passive mobilization Another limitation of this study is the small sample size, which only allowed limited conclusions on the effect of age, sex and BMI on CBFV as covariates. However, the sample size was big enough to assess the primary outcome of this study, the impact of progressive verticalization on CBFV and was adequate for an observational pilot study (7). The small sample size did not allow adjusting for medications of the participants. Although only used by four participants, antihypertensives could potentially influence cerebral perfusion (22). However, the usage of antihypertensives has a high prevalence, being above 20%, for example in the US (23). Therefore, allowing participants currently taking antihypertensive medication to continue this during the measurements led to a more accurate depiction of the general population.

This study was conceived as the starting point for further investigations in patients with impaired regulation of CBF. It is known, that patients show impairments in regulation of CBF after stroke (1) and that these impairments can be predictive of worse functional outcome (2). While it is known that CBFV changes with different head positions (20), there is little data on CBFV in different body positions post-stroke (24). Furthermore, the ideal time for first mobilization post-stroke remains debated (25). As we show that verticalization according to our protocol is feasible in a standardized way, and that it has many parallels to normal mobilization concerning the circulatory response, this or a similar protocol could be used to study CBF regulation post-stroke. Better understanding the hemodynamic responses during verticalization is crucial for safe and efficient mobilization standards in the early phase after stroke.

## 5. Conclusion

Our study provides evidence for CBF regulation in a dynamic verticalization protocol. Potential mechanisms responsible for the drop in CBFV must take effect quite quickly, at least in the healthy participants analyzed here. In addition, our study shows that in healthy subjects, CBFV progressively changes with increasing angles of verticalization. In summary, this study shows that a more dynamic verticalization protocol produces similar results regarding CBFV as those with longer durations of stay in healthy subjects and demonstrates that the changes in CBFV happen progressively. Thus, it provides a reference group, to which patients with impairment of CBF regulation can be compared.

## Data availability statement

The raw data supporting the conclusions of this article will be made available by the authors, without undue reservation.

## Ethics statement

The studies involving human participants were reviewed and approved by Cantonal Ethics Committee Zurich, Switzerland. The patients/participants provided their written informed consent to participate in this study.

## Author contributions

JH, JV, and AS organized and planned the study. JH, TP, AS, and AL recruited participants and acquired the data. TP applied the TCD device to the subject and oversaw data recording. JD did the data analysis. JD, JH, SW, and CG wrote and edited the manuscript. JH, AS, JV, SW, AL, ARL, and CG were responsible for critically revising the manuscript. All authors approved the final draft of the manuscript.
